# A humanized mouse model for sequestration of *Plasmodium falciparum* sexual stages and *in vivo* evaluation of gametocytidal drugs

**DOI:** 10.1038/srep35025

**Published:** 2016-10-12

**Authors:** Yoann Duffier, Audrey Lorthiois, Pau Cisteró, Florian Dupuy, Grégory Jouvion, Laurence Fiette, Dominique Mazier, Alfredo Mayor, Catherine Lavazec, Alicia Moreno Sabater

**Affiliations:** 1 Inserm U1016, Institut Cochin, Paris, France; 2 Cnrs, UMR8104, Paris, France; 3 Université Paris Descartes, Sorbonne Paris Cité, Paris, France; 4 Laboratoire d’excellence GR-Ex, Paris, France; 5 ISGlobal, Barcelona Ctr. Int. Health Res. (CRESIB), Hospital Clínic - Universitat de Barcelona, Barcelona, Spain; 6 Human Histopathology and Animal Models, Institut Pasteur, Paris, France; 7 Sorbonne Universités, UPMC Univ Paris 06, INSERM U1135, CNRS ERL 8255, Centre d’Immunologie et des Maladies Infectieuses (CIMI-Paris), 91 Bd de l’hôpital, F-75013, Paris, France; 8 AP-HP, Groupe hospitalier La Pitié-Salpêtrière, Service de Parasitologie Mycologie, F-75013, Paris, France; 9 Genetics and Genomics of Insect Vectors Unit, Institut Pasteur, Paris, France; 10 AP-HP, Hôpital St Antoine, Service de Parasitologie-Mycologie, Paris, France

## Abstract

The development of new drugs to disrupt malaria transmission requires the establishment of an *in vivo* model to address the biology of *Plasmodium falciparum* sexual stages (gametocytes). Herein we show that chemically immune-modulated NSG mice grafted with human erythrocytes support complete sexual development of *P. falciparum* parasites and generate high gametocytemia. Immunohistochemistry and RT-qPCR analyses indicate an enrichment of immature gametocytes in the bone marrow and the spleen, suggesting a sequestration mechanism reminiscent to that observed in humans. Upon primaquine treatment, elimination of gametocytes from peripheral blood and from sequestration sites was observed, providing a proof of concept that these mice can be used for testing drugs. Therefore, this model allows the investigation of *P. falciparum* sexual commitment, gametocyte interactions with the bone marrow and spleen and provides the missing link between current *in vitro* assays and Phase I trials in humans for testing new malaria gametocytidal drugs.

The eradication of malaria requires the development of new transmission-blocking drugs that have to be tested in animal models^1^. *Plasmodium falciparum* transmission from humans to mosquitoes is ensured by the parasite sexual stages called gametocytes. Immature gametocytes, from stage I to IV, develop in erythrocytes that sequester approximately 10 days in internal organs. Only the mature stages (stage V) are found in the peripheral blood where they are available for ingestion by mosquitoes. Recent molecular and histological studies of post-mortem specimens and clinical studies from infected individuals revealed that immature gametocytes are present in the extravascular compartment of the human bone marrow^2^,^3^,^4^. In addition, early post-mortem observations and a recent clinical case report on a splenectomized patient suggested that immature gametocytes might also sequester in the spleen^5^,^6^,^7^. However, it is still unclear whether the spleen is a site for maturation or clearance of immature gametocytes. The molecular mechanisms underlying the sequestration of gametocytes, followed by their release upon maturation into the circulation, remains one of the unanswered questions in the biology of malaria parasites that needs to be addressed by *in vivo* studies^8^.

One of the major challenges in studying *P. falciparum* gametocytes in laboratory animals is the parasite’s specificity for its human host and the important biological differences that exist between *P. falciparum* and rodent malaria gametocytes. Moreover, the use of non-human primates is limited due to economic and ethical considerations. In addition, *P. falciparum* infection with a gametocyte-producing parasite strain can only be achieved in splenectomized animals^9^,^10^, precluding the use of monkeys to address gametocytes’ interactions with the spleen. Consequently, mechanisms underlying *P. falciparum* gametocytogenesis have never been addressed *in vivo*. In addition, the lack of appropriate experimental models has restricted the evaluation of new transmission-blocking drugs to *in vitro* assays, which do not account for factors such as drug metabolism or gametocyte sequestration that might complicate intervention approaches. The generation of mouse strains with severe immunodeficiency and grafted with human red blood cells (hRBC) has allowed the establishment of humanized mouse models for *P. falciparum* erythrocyte infection that are currently being used to test anti-malarial drugs that target asexual parasites^11^,^12^. Further development of transgenic immune-deficient mice has made it possible to study the parasite pre-erythrocytic cycle and the complete life cycle was obtained in mice co-grafted with human hepatocytes and hRBC^13^,^14^. However, parasite sexual development in these humanized mouse models is still challenging due to the high turnover rate of infected hRBC, which is not optimal for the complete maturation of gametocytes. A few authors have reported presence of gametocytes in peripheral blood of *P. falciparum*-infected humanized mice, however gametocytes were only detected at very low levels (below 0.001%)^13^,^15^,^16^. High gametocytemia have only been reported when mouse splenic function was reduced by splenectomy^17^ or by using an immunomodulation protocol that led to gametocyte formation and maturation in the peritoneum^11^,^18^. Consequently, these models have not been developed further and to date there are no reports describing their usefulness to test the ability of compounds to eliminate *P. falciparum* gametocytes, nor their relevance as a model to address gametocyte interactions with the bone marrow and the spleen.

To overcome the limitations of these humanized models, we optimized an immunosuppression protocol to decrease the macrophage load in the spleen and liver^18^, thereby increasing the half-life of grafted hRBC and allowing gametocyte sequestration in internal organs. We applied this protocol to the severe immune-deficient mouse strain NOD SCID gamma c (NSG) and followed gametocyte development and distribution in different mice organs after *P. falciparum* infection.

## Results and Discussion

**Figure 1:**
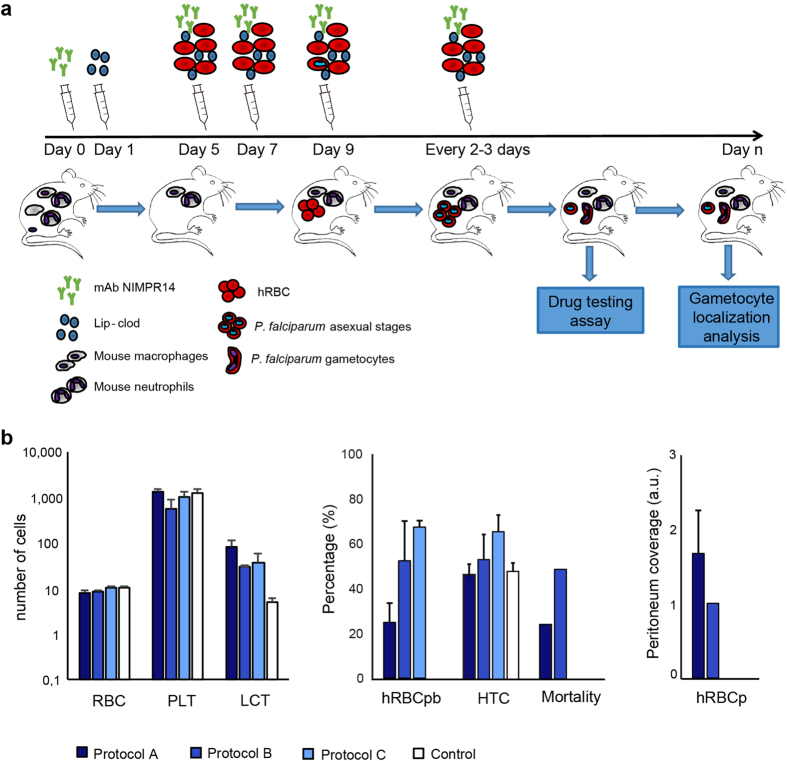
Establishment of a new protocol for *Plasmodium falciparum* sexual development in humanized mouse. (**a**) Experimental procedure for engraftment of NSG mice with hRBC before infection with *P. falciparum* parasites. (**b**) Hematological parameters and mortality in 4 groups of mice (n = 4 mice per group) treated with different immunomodulation protocols. Protocol A: 33.3 mg/kg of lip-clod +10 mg/kg of mAb NIMP-R14, protocol B: 13.3 mg/kg of lip-clod +2 mg/kg of mAb NIMP-R14 and protocol C: 6.25 mg/kg of lip-clod +1 mg/kg of mAb NIMP-R14. Control mice received RPMI 1460 medium. Measurement of total RBC (mouse and human RBC, 10^6^/mm^3^), platelets (PLT, 10^3^/mm^3^), leucocytes (LCT, 10^3^/mm^3^), hematocrit (HTC, %) and percentage of hRBC (hRBCpb) in mice peripheral blood samples were performed by using an automatic hematology analyzer at day 9 of the protocol. Mortality was recorded during 9 days and surviving mice (4 in Control group, 3 in group A, 2 in group B and 4 in group C) were sacrificed at day 9. Presence of hRBC in peritoneum (hRBCp) was determined after sacrifice. Complete coverage of this cavity with hRBC was noted as 2 points, partial coverage was noted as 1 point and absence of hRBC was noted as 0 point.

**Figure 2:**
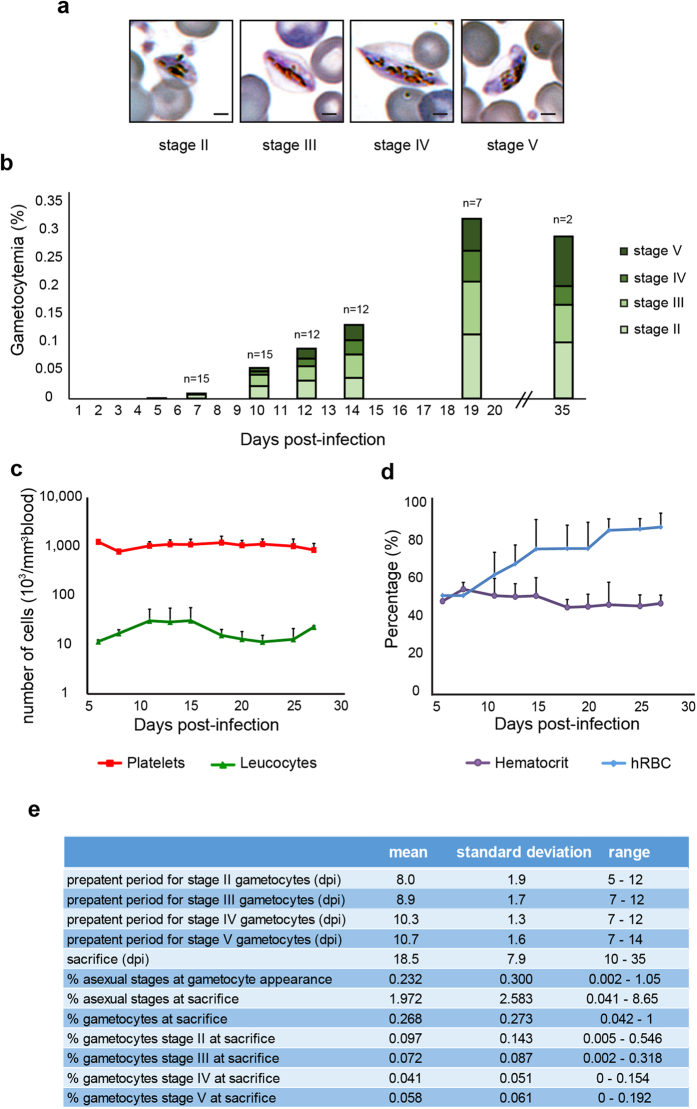
*P. falciparum* gametocytes develop in humanized NSG mice. (**a**) Representative pictures of stage II, III, IV and V gametocytes in peripheral blood as observed in Giemsa-stained thin blood smears. Bars represent 2 μm. (**b**) Kinetics of gametocytemia for stage II, III, IV and V gametocytes in peripheral blood during 35 days of infection. Results represent the average gametocytemia of 15 mice from 4 independent infection experiments. Mice were sacrificed at different time points (3 mice at day 10, 5 mice at day 14, 5 mice at day 19 and 2 mice at day 35). (**c,d**) Follow up of hematological parameters in 8 mice during gametocyte development. Measurement of platelets, leucocytes, hematocrit and % of hRBC in mice peripheral blood samples were performed with an automatic hematology analyzer. (**e**) Kinetics of asexual stages and gametocytes in peripheral blood during the course of infection. Parasitemia, gametocytemia (% of parasites in total RBCs) and prepatent period (time between inoculation and detection of parasites on thin blood smears) for each gametocyte stage were determined by counting parasites on Giemsa-stained thin blood smears. Data represent the average of 15 mice from four independent infection experiments. dpi: days post-infection.

In previous reports we have shown that an immunomodulation protocol in immune-deficient mice allows the engraftment of hRBC and subsequent *P. falciparum* infection by intraperitoneal route^18^,^19^. In this protocol, the depletion of neutrophils and macrophages in the peritoneum, the spleen and the liver was induced by NIMP-R14 mAb and by clodronate encapsulated in liposomes (lip-clod), respectively. However, the high concentrations of NIMP-R14 mAb and lip-clod used in this protocol resulted in hRBC retention in the mouse peritoneum and consequently a high proportion of hRBC did not reach the peripheral blood, leading to gametocyte formation and maturation in the peritoneum^11^,^18^. This artificial model did not reflect *P. falciparum* gametocyte development in humans and precluded its use to address gametocyte interactions with host organs. To improve hRBC engraftment in peripheral blood, we optimized a new immunosuppression protocol in the severe immune-deficient mouse strain NOD SCID gamma c (NSG) (Fig. 1a). This mouse strain lacks mature T cells, B cells and Natural Killer cells, and is an excellent recipient mouse model for engraftment of human cells^15^,^20^. To overcome hRBC retention in mouse peritoneum, we tested three different combinations of lip-clod and mAb NIMP-R14 (Fig. 1b). Our results showed that the combination of 6.25 mg/kg of lip-clod, 1 mg/kg of NIMPR-14 and 0.75 mL of hRBC allowed the graft of human cells in peripheral blood without residual retention in peritoneum. This combination also reduced mouse mortality without inducing any major changes in other hematological parameters (Fig. 1b). Eighteen NSG mice were humanized with this immunomodulation protocol and infected with *P. falciparum* asexual parasites in four independent experiments (Fig. 1a). The parasitemia and gametocytemia were monitored daily in thin tail-blood smears from day 3 post infection (dpi) until sacrifice at different time points. Asexual parasites were detected in the peripheral circulation of the 18 mice and gametocytes at different stages of maturity (stage I to V) were observed in 15 mice (Fig. 2a), with peaks of gametocytemia ranging from 0.042% to 1% (Fig. 2b,e). During the course of gametocyte development, engraftment of hRBC in peripheral blood increased from 50% to 85% whereas other hematological parameters remained stable (Fig. 2c,d). Appearance of stage II gametocytes in the peripheral blood occurred on average 8 days after injection of asexual parasites and stage I gametocytes were still observed up to 35 days post-infection (Fig. 2e). These results indicate that parasite sexual commitment occurred in mice rather than *in vitro* prior to injection, thus supporting the use of this humanized mouse model to address *in vivo* the mechanisms underlying gametocytogenesis.

**Figure 3:**
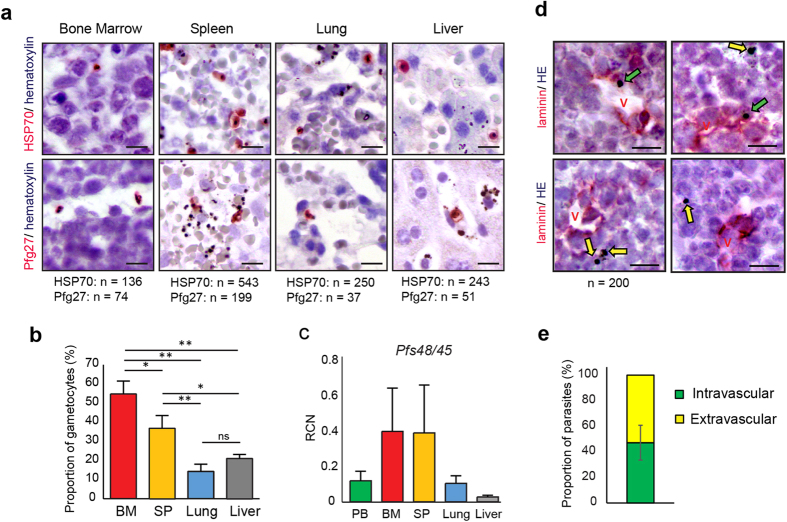
Gametocytes are enriched in the bone marrow and the spleen. (**a**) Histological sections of sternum bone marrow, spleen, liver and lung were stained with antibodies directed against Pfg27 and HSP70 to detect gametocytes and all parasites, respectively. n = total number of HSP70- and Pfg27-positive parasites observed in 30 fields at 630X magnification for each tissue sample for 5 mice (a total of 1, 200 fields was analyzed). Bars represent 10 μm. (**b**) Proportion of Pfg27-positive gametocytes in the HSP70-positive parasite population in bone marrow (BM), spleen (SP), lung and liver. Results represent the average ratio of Pfg27-positive cells in Pfg27-stained sections to HSP70-positive cells in HSP70-stained sections observed in 30 fields. Stars represent significant differences in proportion (**P < 0.01, *P < 0.05). ns: not significant. (**c**) Quantitative analysis by real time RT-qPCR of gametocytes distribution in peripheral blood (PB), femur bone marrow (BM), spleen (SP), lung and liver. Relative copy numbers (RCN) of the gametocyte marker *Pfs48/45* were calculated as the ratio between copy numbers of *Pfs48/45* transcripts and the geometric mean of the ring upregulated gene *sbp1* and the trophozoite upregulated gene *hprt* transcripts. Results represent the average quantification for 8 mice from two independent infection experiments. (**d**) Histological sections of bone marrow (sternum) were stained with antibodies directed against laminin to detect basement membrane of microvasculature (V). Hemozoin malaria pigment allows detection of parasites in intra- (green arrow) or extra- (yellow arrow) vascular space. Bars represent 10 μm. (**e**) Proportion of intra- and extra-vascular parasites observed in a population of 200 parasites on laminin-stained histological sections from three mice.

To evaluate whether this new immunosuppression protocol in NSG mice allows the parasite to sequester and mature in the spleen and bone marrow, we investigated the parasite distribution in different mouse organs. Immunohistochemical labeling was performed on samples of bone marrow, spleen, liver and lung of 5 mice with antibodies against the constitutively expressed heat shock protein 70 (HSP70), that detects all parasites, or the gametocyte specific antigen Pfg27 that detects stage I to stage V gametocytes^21^ (Fig. 3a). The proportion of gametocytes in the parasite population was 2 to 3-fold higher in the bone marrow and spleen than in the lung and liver (Fig. 3b). To further confirm this observation, we performed a real-time RT-qPCR analysis to measure gene expression of the gametocyte specific gene *Pfs48/45* related to the asexual stage specific genes *hypoxanthine phosphorybosyltransferase* (*hprt*) and *skeleton binding protein 1* (*sbp1*) in peripheral blood, bone marrow, spleen, liver and lung of 8 mice (Fig. 3c). The mean expression of *Pf48/45* was about 3-fold higher in the bone marrow and spleen compared to peripheral blood, lung and liver. This indicates that gametocytes were more abundant in the bone marrow and spleen than in peripheral blood and other organs, in agreement with the immunohistochemical analysis. To determine the spatial distribution of parasites within the bone marrow, sternum bone marrow sections were stained with anti-laminin antibody to label the basement membrane of the host vasculature^22^ and hemozoin malaria pigments were localized relative to the laminin-positive microvasculature (Fig. 3d). In three mice with 54–65% gametocytes in their bone marrow, as determined by the ratio Pfg27/HSP70, we observed an average of 47% of parasites associated with laminin and 53% in the extravascular space (Fig. 3e). Although presence of hemozoin pigments is not specific for gametocytes, these observations suggest that a proportion of gametocytes were enriched in the extravascular compartment of bone marrow, as observed in humans^4^.

**Figure 4:**
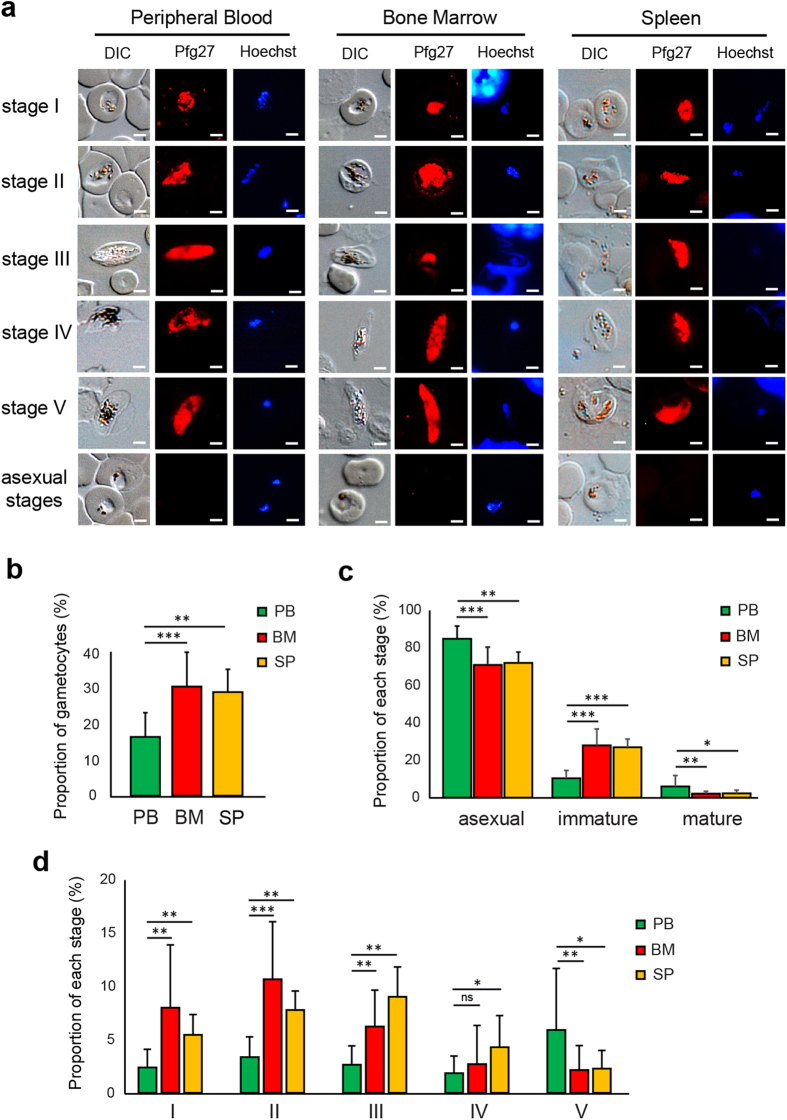
Immature gametocytes are enriched in the bone marrow and the spleen. (**a**) The proportion of gametocytes in peripheral blood, femur bone marrow aspirate or spleen extract was analyzed by immunostaining and determined by calculating the percentage of Pfg27-positive parasites in the total parasite population detected by Hoechst staining. Different developmental stages were determined by analysis of gametocyte morphology on differential interference contrast images. Bars represent 2 μm. (**b**) Average proportion of gametocytes in the parasite population observed in peripheral blood (PB), bone marrow (BM) and spleen (SP). PB: 311 gametocytes observed in 12 mice, BM: 213 gametocytes observed in 12 mice, SP: 123 gametocytes observed in 6 mice. (**c**) Average proportion of asexual stages, immature gametocytes and mature gametocytes in the parasite population observed in PB, BM and SP. (**d**) Average proportion of each gametocyte stage in the parasite population observed in PB, BM and SP. (**b–d**) Results represent the average proportion for 12 mice (PB and BM) or 6 mice (SP) from four independent infection experiments. Stars represent significant differences in proportion (***P < 0.001, **P < 0.01, *P < 0.05). ns: not significant. Graphs were generated using GraphPad Prism software. Error bars represents Standard Error of the Mean (SEM).

To address the distribution of each gametocyte stage in the bone marrow and spleen compared to peripheral blood, we performed an immunostaining analysis using an antibody against the gametocyte marker Pfg27 on smears of peripheral blood, bone marrow aspirate, and spleen extract (Fig. 4a). Results showed that only immature gametocytes from stage I to III were significantly enriched in the bone marrow and spleen, whereas mature gametocytes were more abundant in the peripheral blood (Fig. 4b–d). These results suggest that immature gametocytes sequester in the bone marrow and spleen of these mice and are released into the circulation upon maturation, although enrichment of gametocytes in the spleen may also reflect their retention in splenic slits before clearance by macrophages. In either case, our results are in accordance with observations made in humans^2^,^4^,^7^. However, the observation that one third of the immature gametocyte population is present in the peripheral circulation suggests that the sequestration mechanism occurring in this mouse model is incomplete. A possible explanation is that gametocyte sequestration in humans may be due to a combination of cytoadhesion and mechanical retention mechanisms, and the lack of human specific receptor(s) in NSG mice might only allow rigidity-mediated sequestration due to the important stiffness of immature gametocyte-infected erythrocytes^23^. The development of a long-term humanized mouse model with human bone marrow transplantation would allow to decipher the putative respective roles of cytoadhesion and mechanical retention in sequestration mechanisms. Altogether these exciting observations indicate that this humanized mouse model provides a unique opportunity to address *in vivo* the mechanisms underlying sequestration of immature gametocytes in human bone marrow and potentially in the spleen.

**Figure 5:**
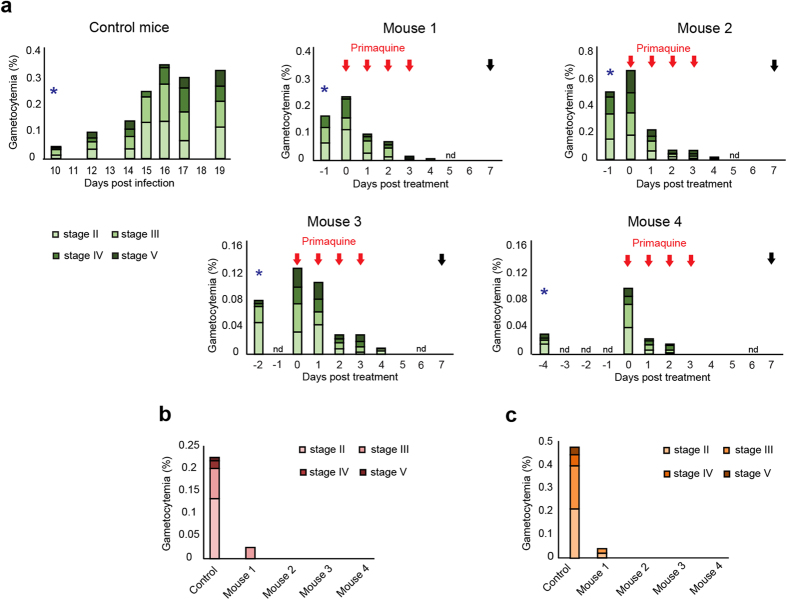
Humanized NSG mice provide a suitable model for testing malaria gametocytidal drugs. (**a**) Kinetics of gametocytemia for stage II, III, IV and V gametocytes in peripheral blood in 10 untreated mice (Control mice) and in 4 primaquine-treated mice during 8 to 11 days following appearance of gametocytes in peripheral blood (blue star). Gametocytemia (% of gametocytes in total RBCs) for each gametocyte stage were determined by counting parasites on Giemsa-stained thin blood smears. Primaquine treatment (2 mg/kg) was started 1 to 4 days after appearance of gametocytes and was daily administered for 4 days (red arrows). Mice were sacrificed seven days after beginning of treatment (black arrow). (**b**) Gametocytemia in bone marrow after sacrifice as observed in Giemsa-stained smears of bone marrow aspirate. Control represents an average of gametocytemia for 10 mice from three independent infection experiments. (**c**) Gametocytemia in spleen after sacrifice as observed in Giemsa-stained smears of crushed spleen. Control represents the average gametocytemia of 6 mice from two independent infection experiments.

We next explored the potential of the NSG mice model as a platform for testing drugs against *P. falciparum* transmission stages. Indeed, humanized mice have a crucial role for drug discovery in malaria, as they offer the opportunity to assess *in vivo* the activity of drugs. As a proof of concept, we studied the effect of primaquine, which is currently the only licensed drug that has demonstrated efficacy to eliminate *P. falciparum* gametocytes in humans^24^. A group of 4 mice were treated with primaquine (2 mg/kg; intraperitonealy) for 4 days, starting 1 to 4 days after appearance of *P. falciparum* gametocytes in peripheral blood (Fig. 5a). In the 4 mice, with gametocytemia ranging from 0.1% to 0.66% at day 0, gametocytes of all stages were totally eliminated from peripheral blood after 3 to 6 days of treatment. In contrast, in 10 untreated mice from 3 independent infection experiments, the average gametocytemia increased from 0.045% to 0.325% during the 9 days following appearance of gametocytes in peripheral blood. As expected, asexual stages were also eliminated from peripheral blood after 3 to 6 days, since primaquine treatment at 2 mg/kg represents a dose 1000-fold upper the IC50 of primaquine on asexual stages^25^,^26^ (Supplemental Figure S1). In addition, the efficacy of primaquine to eliminate gametocytes from sequestration sites was evaluated in Giemsa-stained smears of bone marrow aspirates and spleen extracts and compared to the average of gametocytemia in 10 untreated mice (Fig. 5b,c). Gametocytes were totally eliminated from both organs in 3 treated mice, whereas one mouse still harbored a significant gametocytemia, corresponding to 10% (in bone marrow) and 16% (in spleen) of the initial gametocytemia in peripheral blood at day 0 of primaquine treatment. These data show that the immunosuppression protocol used to engraft mice does not interfere with the assessment of gametocytidal treatments and provides the proof of concept that this model is suitable for testing new drugs against *P. falciparum* transmission stages. Importantly, this protocol can be used to address the effect of a drug on the persistence of gametocytes in their sequestration sites, which is crucial information that could not be provided by clinical trials in humans.

In summary, these humanized mice are a suitable model to investigate *P. falciparum* sexual commitment *in vivo*, gametocyte interactions with the bone marrow and the spleen, and they may facilitate the discovery of new gametocytidal drugs, which is urgently needed to achieve the goal of malaria eradication.

## Methods

### Ethics statements

This study was carried out in strict accordance with the guide for the care and use of laboratory animals from the Centre d’Expérimentation Fonctionnelle (CEF, La Pitié-Salpêtrière, Paris) and with the French and European regulations (2010/63/EU). The experimental protocols were approved by the Ministère de l′Education Nationale, de l’Enseignement Supérieur et de la Recherche (Authorization Number 01737.03).

### Immunomodulation treatment and mouse infection

NSG mice aged 9–11 weeks (Charles River, US) were bred at the CEF under strict pathogen-free conditions. They were provided with UV light-exposed commercial food and autoclaved water *ad libitum*. Human red blood cells (hRBC) were obtained from donors without history of malaria (Etablissement Français du Sang Ile-de-France, Rungis). Before peritoneal injection into mice, hRBC were washed twice with RPMI-1640 medium (Gibco/BRL) at 900 g, 10 min at 25 °C. The depletion of tissue macrophages was induced by clodronate (Roche Diagnostics) encapsulated in liposomes (lip-clod) as described^27^. Neutrophils were depleted using the monoclonal antibody (mAb) NIMP-R14 produced by a hybridoma kindly provided by Dr M. Strath (National Institute for Medical Research, London, U.K.)^28^. To obtain the graft of hRBC and subsequent *P. falciparum* NF54 infection, each mouse received by intraperitoneal injection a dose of 1 mg/kg of mAb NIMP-R14 at day 0 and 6.25 mg/kg of lip-clod at day 1. At day 5 and day 7 each mouse received 1.5 mL of hRBC at 50% hematocrit in RPMI mixed with 1 mg/kg of mAb NIMP-R14 and 6.25 mg/kg of lip-clod. At day 9 each mouse received the same doses of immunomodulators in 1.5 mL of hRBC at 50% hematocrit containing 5.10e^5^ *P. falciparum* NF54 asexual parasites. Parasites used for infection were obtained from an *in vitro* culture maintained below 1.5% parasitemia to avoid *in vitro* induction of gametocytogenesis. After *P. falciparum* infection, mice were grafted with 1 to 1.5 mL of hRBC at 50% hematocrit containing 1 mg/kg of mAb NIMP-R14 and 6.25 mg/kg of lip-clod every 2–3 days. Hematological parameters (hematocrit, leucocytes, platelets, percentage of hRBC in peripheral blood) were followed up during the assay in blood samples taken from mouse tails and analyzed with an automatic hematology analyzer (Scil Vet abc Plus, Scil Animal Care). Mice exhibiting hematocrit up to 60% and percentage of hRBC higher than 70% only received the graft of hRBC once the hematocrit decreased to 50%. Presence of residual hRBC in the peritoneum was assessed after sacrifice of the mice.

### RT-qPCR analysis

Peripheral blood samples and femur bone marrow aspirate samples were added to Trizol (Life technologies) and vortexed. Spleen, liver and lung sections were processed through grinding in Trizol. RNA was prepared using the PureLink RNA Mini kit (Life technologies) and treated using *on-column* DNase-Treatment with Pure Link DNase (Life technologies). Quantity and purity of RNA were assessed with Epoch spectrophotometer. Contamination with genomic parasite DNA was assessed by qPCR with primers for *P. falciparum seryl-tRNA synthetase* (*PF07_0073*) and positive samples were retreated with DNAse and retested for lack of genomic DNA. RNA (100–200 ng for peripheral blood and total RNA for other organs) was used for complementary DNA (cDNA) synthesis with the SuperScript III First-Strand Synthesis System (Life technologies). Positive cDNA synthesis was confirmed by qPCR targeting *P. falciparum seryl-tRNA synhtetase*. Primers for the gametocyte marker *Pfs48/45* (*PF13_0247*)^2^, the trophozoite upregulated gene *hprt* (*PF10_0121*)^29^ and the ring upregulated gene *sbp1* (*PFE0065w*)^29^ were used to detect transcripts from gametocytes, trophozoites and rings, respectively (Supplemental Table S1). Final reaction volumes of 20 uL included 4 uL of cDNA (3–30 ng/uL) and 10 uL of Power SYBRGreen Master Mix (Applied Biosystems). qPCRs were performed in a ABi Prism 7500 (Applied Biosystems) with 2 minutes at 50 °C, 10 minutes at 95 °C (1 cycle), 30 seconds at 95 °C, and 1 minute at 60 °C (40 cycles). Each sample was analyzed in triplicate together with a serial dilution of 3D7 gDNA for absolute quantification of transcript copy numbers. To normalize for the level of parasitemia and amount of cDNA loaded in each reaction, relative copy numbers (RCN) were calculated as the ratio between copy numbers of *Pfs48/45* transcripts and the geometric mean of *sbp1* and *hprt* transcripts.

### Immunofluorescence assays

Thin smears of peripheral blood, femur bone marrow aspirate or spleen extract were air-dried and fixed for 10 minutes in methanol chilled at −20 °C. Samples were incubated in 2% bovine serum albumin in PBS for 1 h at room temperature to block non-specific binding. The preparations were then incubated overnight at 4 °C with a rabbit polyclonal anti-Pfg27 antibody^30^ diluted 1/1000 and with AlexaFluor 594-conjugated goat anti-rabbit antibody (Molecular Probes) for 1 hour. Nuclei were stained with Hoechst 33342. Samples were observed at 1000X magnification using a Leica DM 5000 B. At least 50 fields at 1000X magnification were analyzed for each sample.

### Histology and immunohistochemistry

Samples were fixed in 10% neutral buffered formalin, embedded in paraffin and 4 μm-thick sections were cut and stained with standard Hematoxylin-Eosin staining. In order to detect parasites, immunohistochemistry analyses were carried out using a mouse monoclonal anti-*Plasmodium* HSP70 antibody^31^ at dilution 1/1000 and a rabbit polyclonal anti-Pfg27 antibody^30^ at 1/2000. Gametocyte enrichment in different organs was calculated as the ratio of Pfg27-positive cells in Pfg27-stained sections to HSP70-positive cells in HSP70-stained sections observed in 30 fields at 630X magnification for each tissue sample. To determine the location of parasites, indirect labeling of blood vessels was carried out using a rabbit anti-laminin polyclonal antibody (Sigma Aldrich, dilution 1:100) to detect basement membrane of microvasculature. Positive signal was revealed using Histofine Simple Stain MAX PO (mouse or rabbit depending on the primary antibody; Nichirei Biosciences Inc.) according to the manufacturer’s protocol. Color was developed with 3-Amino-9-EthylCarbazole (AEC chromogen; BD Pharmingen). Samples were observed at 630X magnification using a LEICA DM 400B.

### Drug treatment

Primaquine (Sigma Aldrich) at 2 mg/kg in RPMI-1640 (Gibco, BRL) was administered daily by intraperitoneal injection during 4 days. Primaquine treatment was started 1 to 4 days after appearance of gametocytes in peripheral blood. For each mouse, gametocytemia was monitored in thin tail-blood smears collected daily until sacrifice at day 7 post drug treatment.

### Statistical analysis

Statistical significance for differences in gametocyte proportions in different organs was established using Student’s t-test and Wilcoxon Mann-Whitney rank sum test.

## Additional Information

**How to cite this article**: Duffier, Y. *et al*. A humanized mouse model for sequestration of *Plasmodium falciparum* sexual stages and *in vivo* evaluation of gametocytidal drugs. *Sci. Rep*. **6**, 35025; doi: 10.1038/srep35025 (2016).

## Supplementary Material

Supplementary Information
