# Supplementary material for: A humanized mouse model for sequestration of Plasmodium falciparum sexual stages and in vivo evaluation of gametocytidal drugs
Source: Sci Rep. 2016 Oct 12;6:35025. doi: 10.1038/srep35025 (PMC5059736; doi:10.1038/srep35025)
Supplement: Supplementary Information [file srep35025-s1.pdf]

**A humanized mouse model for sequestration of *Plasmodium falciparum* sexual stages and *in vivo* evaluation of gametocytidal drugs**

Yoann Duffier, Audrey Lorthiois, Pau Cisteró, Florian Dupuy, Grégory Jouvion, Laurence Fiette, Dominique Mazier, Alfredo Mayor, Catherine Lavazec, Alicia Moreno Sabater

## Supplemental tables

### Table S1. Primers used for RTqPCR analysis:

| *Gene* | Accession number | Primer forward | Primer reverse |
|---|---|---|---|
| ***Transmission-blocking antigen precursor (Pfs48/45)*** | PF13_0247 | GTAAGCCTAGCTCTTTGAATAGTGA | GACCTACGTTCACGCATATCTGGCT |
| ***Pf ubiquitin-conjugating enzyme transcript*** | PF08_0085 | GGTGTTAGTGGCTCACCAATAGGA | GTACCACCTTCCCATGGAGTATCA |
| ***Trophozoite up-regulated gene hypoxanthine phosphorybosyltransferase*** | PF10_0121 | GTTGCCATCGCTTGTCTTTT | TTCCCTCATCATTAACCAAACA |
| ***Ring upregulated gene skeleton binding protein 1 (sbp1)*** | PFE0065w | GGCACTTGCAACTACCGAA | GCTTGAAAAACCGTCATCGT |

## Supplemental Figures and Legends

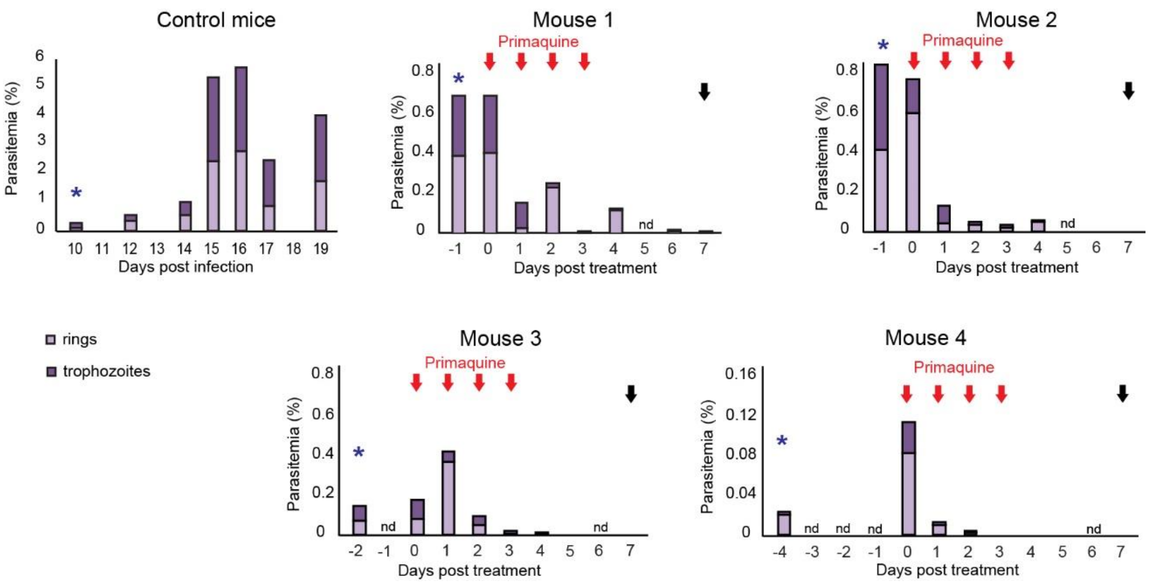


**Supplemental Figure 1: Kinetics of asexual parasitemia in primaquine-treated humanized mice**

Kinetics of asexual parasitemia in peripheral blood in 10 untreated mice (Control mice) and in 4 primaquine-treated mice during 8 to 11 days following appearance of gametocytes in peripheral blood (blue star). Parasitemia (% of rings and trophozoites in total RBCs) was determined by counting parasites on Giemsa-stained thin blood smears. Primaquine treatment (2 mg/kg) was started 1 to 4 days after appearance of gametocytes and was daily administered for 4 days (red arrows). Mice were sacrificed seven days after beginning of treatment (black arrow).
